# Anisotropic multi-step etching for large-area fabrication of surface microstructures on stainless steel to control thermal radiation

**DOI:** 10.1088/1468-6996/16/2/025001

**Published:** 2015-03-17

**Authors:** M Shimizu, T Yamada, K Sasaki, A Takada, H Nomura, F Iguchi, H Yugami

**Affiliations:** 1 Department of Mechanical Systems and Design, Graduate School of Engineering, Tohoku University, Japan; 2 Business Creation Department, Research & Development Division, Dexerials Corporation, Japan

**Keywords:** thermal radiation, wet etching, inteference lithography

## Abstract

Controlling the thermal radiation spectra of materials is one of the promising ways to advance energy system efficiency. It is well known that the thermal radiation spectrum can be controlled through the introduction of periodic surface microstructures. Herein, a method for the large-area fabrication of periodic microstructures based on multi-step wet etching is described. The method consists of three main steps, i.e., resist mask fabrication via photolithography, electrochemical wet etching, and side wall protection. Using this method, high-aspect micro-holes (0.82 aspect ratio) arrayed with hexagonal symmetry were fabricated on a stainless steel substrate. The conventional wet etching process method typically provides an aspect ratio of 0.3. The optical absorption peak attributed to the fabricated micro-hole array appeared at 0.8 *μ*m, and the peak absorbance exceeded 0.8 for the micro-holes with a 0.82 aspect ratio. While argon plasma etching in a vacuum chamber was used in the present study for the formation of the protective layer, atmospheric plasma etching should be possible and will expand the applicability of this new method for the large-area fabrication of high-aspect materials.

## 1. Introduction

Spectral control of thermal radiation is a promising way to improve efficiency of energy systems. There are several methods to control thermal radiation, such as using materials’ intrinsic properties [1, 2], metal–dielectric coatings [3, 4], and microstructures on the metal surface [5, 6]. Surface microstructures are especially promising for high-temperature usage because just a single material needs to be manipulated, and the cut-off wavelength for thermal radiation is tunable by controlling the microstructure shape and size. High-temperature reliability of microstructures of 2D [7] and 3D photonic crystals [8] over 1000 °C has been recently reported.

Spectrally selective materials that can be used at high temperatures can improve the efficiency of systems, such as concentrated solar power generators and thermophotovoltaic systems. For these systems, microstructures must be fabricated over a large metal surface area. However, fabrication of microstructures on a large metals surface is quite difficult. Several studies for fabrication of microstructures on refractory metal surfaces have been reported [9, 10]. Although these microstructures show high spectral selectivity and thermal resistivity, the technique is not easily adaptable to large-area fabrication. To fabricate large-scale spectrally selective materials, the self-organization property of superalloys has been observed [11], but the techniques resulted in low spectral selectivity due to inhomogeneity of microstructures. Silicon anisotropic etching [12] can also be used to fabricate periodic microstructures, but the types of materials and shapes of fabricated microstructures are limited. A few studies on the large-area fabrication of one-dimensional stripe structures have also been reported [13–15]. However, these structures are one-dimensional grating. Therefore, while enhancement of emission at specific wavelengths was achieved, the emission was polarization dependent.

Herein, we describe a method for the fabrication of periodic microstructures on metal substrates based on a multi-step wet-etching process that enables the anisotropic etching of large-area surfaces. This method consists of three main steps, i.e., resist mask fabrication via photolithography, electrochemical wet etching, and side wall protection. Cylindrical micro-holes with a 0.82 aspect ratio arrayed with hexagonal symmetry were fabricated on a 5 cm diameter stainless steel substrate using this method. In the present study, the side wall protection process using argon (Ar) plasma etching was conducted under a vacuum. If atmospheric plasma etching during the formation of the protective layer can be achieved, this fabrication method will be applicable for the large-area fabrication of high-aspect materials.

## 2. Experimental methodology

**Figure 1.**
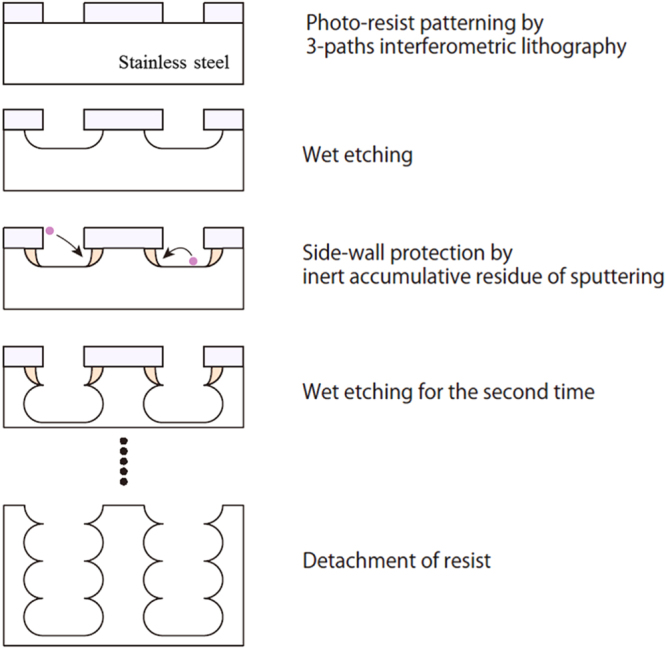
Process flow of the proposed multi-step etching.

In general, dry etching techniques, called Bosch processes [16], are used to fabricate deep micro-holes, because high-accuracy and high-aspect microstructures can be fabricated by this anisotropic etching process, although the size of the fabrication area is limited by vacuum chamber size. On the other hand, wet etching techniques are suited for large-area and low-cost fabrication, but deep microstructures cannot generally be wet etched. To fabricate deep microstructures over a large area, we developed the multi-step wet-etching process as shown in figure 1. First, a patterned resist mask was fabricated by three-beam interference lithography using Mach–Zehnder type interferometer [17]. Because exposure masks and complicated expensive machines are not required for interference lithography, this technique is suitable for large-area fabrication of periodic microstructures [18–20]. Stainless steel SUS304, which is a general austenitic stainless steel, was used as the substrate after polishing. In the lithography process, TDMR-AR80 (Tokyo Ohka Kogyo Co., Ltd) was used for the resist mask, and a Nd:YAG laser with an emission wavelength of 355 nm was used for the exposure. The exposure time was 100 s for an area with a diameter of 5 cm at a laser power of 100 mW. After the fabrication of the resist mask, the first electrochemical wet etching was performed using dilute aqueous 3% oxalic acid, and an applied voltage of 3 V generated with a direct current (dc) power supply. By monitoring the dc between the sample and the counter electrode, the total etched volume was controlled. Throughout the wet etching process, the temperature of the etchant was maintained at 25 °C. After etching, a side wall protective layer was formed via Ar plasma etching (NLD, ULVAC) at a chamber pressure of 0.3 Pa. As shown in figure 1, resist canopies were formed on the tops of the micro-holes due to the isotropic nature of wet etching. These canopies appeared to play a key role in forming the side wall protective layer by preventing the spread of the resist and the oxidized layer of stainless steel residue to the outside of the holes. Thus, due to the presence of the wall protection, the second wet etching process deepened the micro-holes without widening them. Consequently, deep micro-holes could be fabricated by repeating the etching and protection processes. Finally, the resist mask and accumulated inert residue were removed with acetone. As mentioned in section 1, a vacuum plasma etching process was applied in this study. However, it should be possible to use an atmospheric plasma etching process. Therefore, by applying role-to-role processing, this multi-step etching process should be applicable to the large-area fabrication of periodic microstructures.

## 3. Results and discussion

### 3.1. Numerical simulations

**Figure 2.**
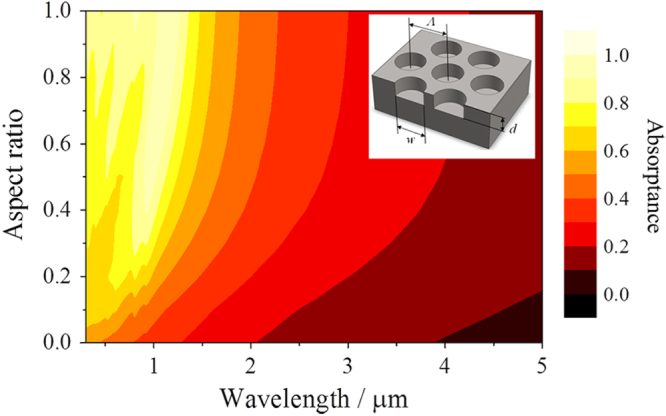
Contour map of simulated absorptance as a function of micro-hole aspect ratio. The inset shows the model parameters for micro-holes arrayed in hexagonal symmetry. The micro-holes width *w* and pitch *Λ* are set to 0.60 and 0.82 *μ*m, respectively.

Enhancement of optical absorption as a function of aspect ratio, which is the ratio of aperture diameter and depth of the micro-holes, was evaluated by numerical simulation based on the rigorous coupled-wave analysis (RCWA) method [21]. The simulation was performed for 2D periodic micro-holes arrayed on a stainless steel substrate, as shown in the inset of figure 2. In the simulation, since it is difficult to measure optical constants for stainless steel, optical constants for iron from literature [22] were used. Simulation results using various aspect ratios for the micro-holes are represented in figure 2. Enhancement of optical absorption at short wavelengths, attributed to the confined effect of micro-holes, is found to be weak when the aspect ratio is lower than 0.3. Optical absorption peak almost reaches 1.0 when the aspect ratio is over 0.8. Therefore, we focused on 0.8 aspect ratios to control the thermal radiation spectrum. A similar result has been seen for a spectrally selective emitter using tungsten 2D microcavities [9].

### 3.2. Multi-step wet etching

**Figure 3.**
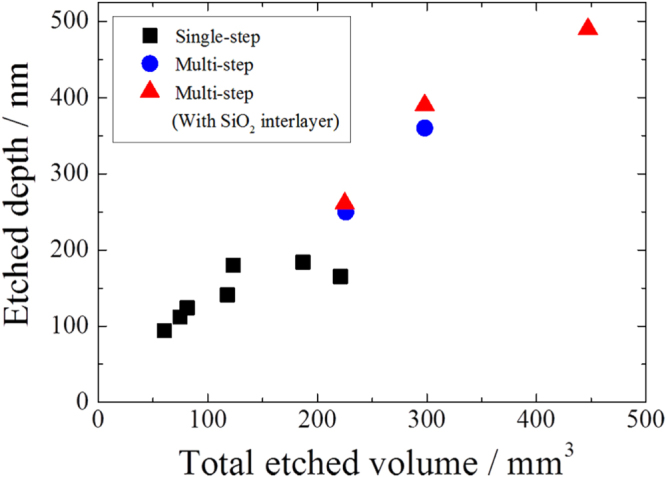
Etched depth of the micro-holes as a function of total etched volume. The black squares, blue circles, and red triangles indicate samples fabricated by single-step etching, multi-step etching, and multi-step etching with SiO_2_ interlayer, respectively.

The multi-step wet etchings explained above are conducted for a 5 cm diameter stainless steel substrate. As shown in figure 3, depth of micro-holes increases with total etched volume according to the following formula:1
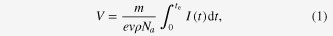
where *m* is atomic weight, *e* is elementary charge, *v* is valence of the ion, *ρ* is density, *t*_e_ is etching time, and *I* is measured current.

With single-step wet-etching processes, etched depths are limited to approximately 180 nm due to isotropic etching. The walls between the holes dissolve from the surface down, which leads to the saturation of the etched depth, and ultimately the holes connect with one another. So in the single-step wet-etching process, the aspect ratio of micro-holes is limited to a maximum of 0.3, which is not sufficient for controlling thermal radiation, as shown in figure 3. The depths of holes were measured by scanning probe microscopy (SPM; SPI-4000, Seiko Instruments Inc.). The measurement was conducted by using a carbon nanofiber probe cantilever.

By our multi-step etching, micro-hole depths exceeded 180 nm and increased steadily to 500 nm, as shown in figure 3. In this process, the amount etched at each step must be carefully controlled to obtain high-aspect ratio micro-holes. By conducting 150 nm etching for 10 min per step, we obtained 150 nm depth holes after the first etching. After the second etching step, holes with a depth of 273 nm (82% of the expected second-step etching depth) were obtained. After third and fourth etchings, we obtained 378 nm depth holes (63% of the expected depth of 600 nm). By reducing the etching rate from 150 to 100 nm, we obtained 360 nm depth holes after the fourth etching (90% of the expected depth of 400 nm). This means that the etching rate gradually decreased with each etching step as micro-hole depths increased, because the etchant rate of the micro-holes stagnated with increasing aspect ratio. Therefore, reduction in the etching volume per step was needed to obtain high-aspect ratio micro-holes.

Further, we increased the micro-hole depths by using a silicon dioxide (SiO_2_) interlayer between the resist mask and substrate. The SiO_2_ interlayer adhesion prevented the etchant from seeping into the crevice between the mask and substrate. As shown in figure 3, after four 100 nm etching steps, the total etched depth increased by 10% compared to that achieved for the sample without a SiO_2_ layer.

In summary, decreasing the etching volume per etching step, increasing the number of etchings, and using a SiO_2_ interlayer enabled the fabrication of micro-holes with a depth of 490 nm and an aspect ratio of 0.82.

**Figure 4.**
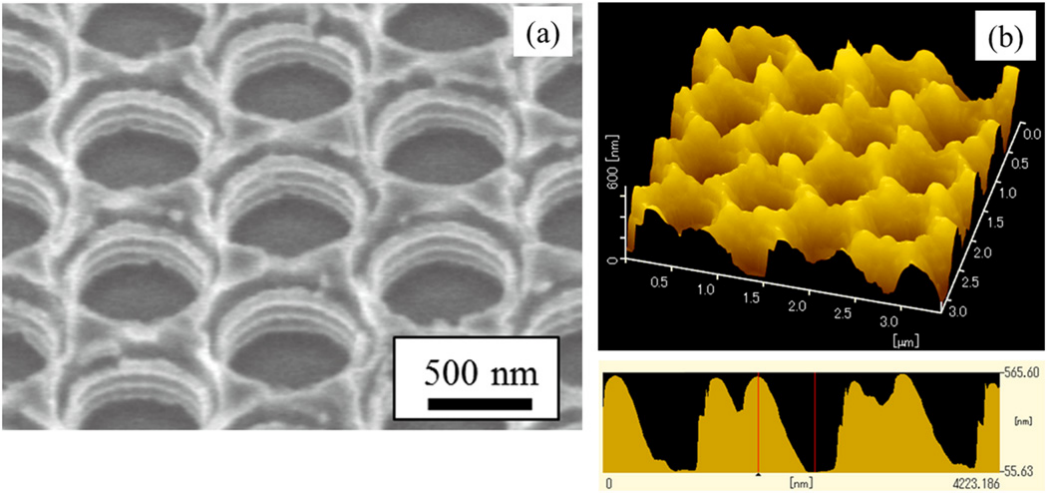
(a) SEM image of the sample after four repeated etching steps using resist and SiO_2_ interlayer as a mask. The etching depth is set to 100 nm for each step. (b) SPM image of the sample after six repeated etching steps using resist and SiO_2_ interlayer as a mask. The depth of 490 nm is obtained.

The micro-holes fabricated by the multi-step etching process are shown in figure 4. A scalloped pattern in the walls of the micro-holes was observed by SEM (figure 4(a)). The depths of the holes can be found in the SPM image shown by figure 4(b). A depth of 490 nm can be obtained by six etching steps.

In this experiment, some processes were conducted under vacuum, such as the SiO_2_ interlayer etching and the wall protection. However, it is possible for these processes to be performed without vacuum. For instance, SiO_2_ interlayers can be electrochemically etched using potassium hydroxide, and the wall protection would be formed by blasting off the inert accumulated residue using atmospheric-pressure plasma.

**Figure 5.**
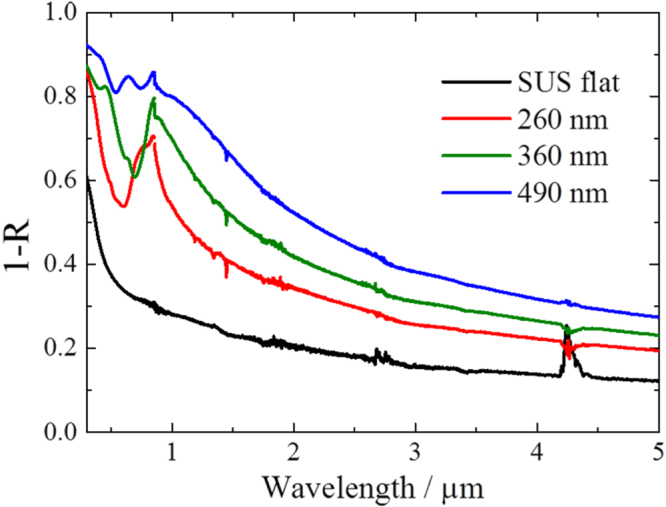
Absorptance spectra calculated from measured reflectance of the etched samples. Red, green, and blue solid lines indicate micro-hole depths of 260, 360, and 490 nm, respectively. The black solid line shows the absorptance spectrum of the non-etched stainless steel substrate.

**Figure 6.**
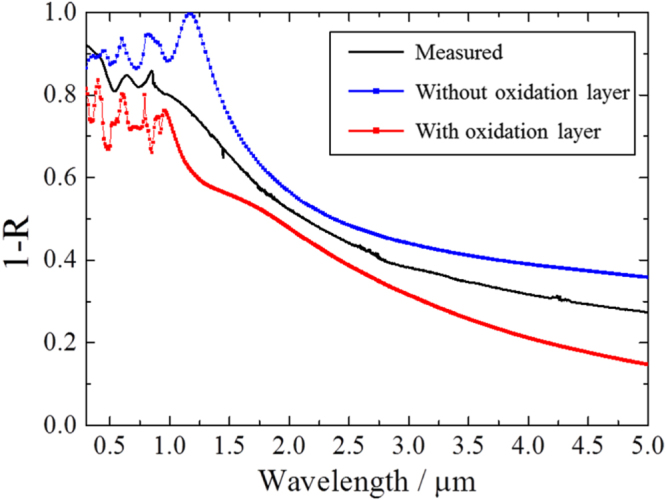
Absorptance spectra of micro-holes with depth 490 nm compared with simulation result using RCWA. The simulated model shows 0.82 aspect ratio and has tapered walls. The blue line with square dots shows the simulation results without an oxide layer, and the red line with square dots represents a model with a 0.3 *μ*m oxide layer.

The hemispherical reflectance spectra of the fabricated samples were measured using a spectrophotometer (Lambda 900; Perkin Elmer) for the visible light range and a Fourier transform infrared spectrophotometer (FT-IR: Spectrum-GX; Perkin Elmer) for the infrared range. Integrating spheres with 15 mm diameter were used for both measurements. The absorptance of the samples calculated from the measured reflectance is shown in figure 5. The optical absorption peak attributed to electromagnetic waves confined by the micro-holes appeared at around 0.8 *μ*m, and the peak shifted to longer wavelengths with increasing depth. The small sharp peak at 0.85 *μ*m did not shift with changing micro-hole depth. Therefore, the small peak seems to be related to the surface coupling mode such as surface plasmon–polariton propagation. The peak attributed to the confined mode broadened and its intensity increased steadily with increasing micro-hole depth. The peak absorptance exceeded 0.8 for the sample with a micro-hole depth of 490 nm and aspect ratio of 0.82, although the simulation indicated the peak absorptance should reach 1.0. As shown in figure 4, oxide material appears to be on the walls and wall tops, judging by the contrast difference in the SEM image. A simulation result taking this factor into consideration is shown in figure 6 as a red line. Changes in reduction in the optical absorption peak and decrease in the absorptance slope at the cut-off wavelength were consistent with the measured result. Therefore, spectral selectivity improved with the removal of the surface oxide material.

## 4. Conclusions

This study focused on the development of a method for the large-area fabrication of periodic microstructures intended to control the thermal radiation spectra of materials. The proposed multi-step wet-etching process enables the fabrication of periodic microstructures on metal substrates with comparatively high-aspect ratios. An aspect ratio of 0.82 was obtained by protecting the side walls from the resist and substrate surface residue scattered during plasma etching. In this study, Ar plasma etching under a vacuum was used for the protective layer formation. However, an atmospheric plasma etching process should also be effective, and therefore, this method should be compatible with large-area fabrication processes, such as role-to-role processes. The material fabricated on stainless steel exhibited highly selective absorption with high absorbance (>0.8) in the visible light and near-infrared regions and low absorbance at longer wavelengths (<0.3). Therefore, this method is expected to advance the practical use of spectrally selective materials in high temperature applications and corrosive environments and as solar selective absorbers, infrared sources for gas sensors, and so on.
